# Supplementary figures and images for: Single-Cell Lineage Trajectory Defines Cyclin-Dependent Kinase Inhibitor–Sensitive Cells-of-Origin in Esophageal Squamous Cell Carcinoma
Source: Gastro Hep Adv. 2025 Dec 29;5(3):100874. doi: 10.1016/j.gastha.2025.100874 (PMC12865637; doi:10.1016/j.gastha.2025.100874)

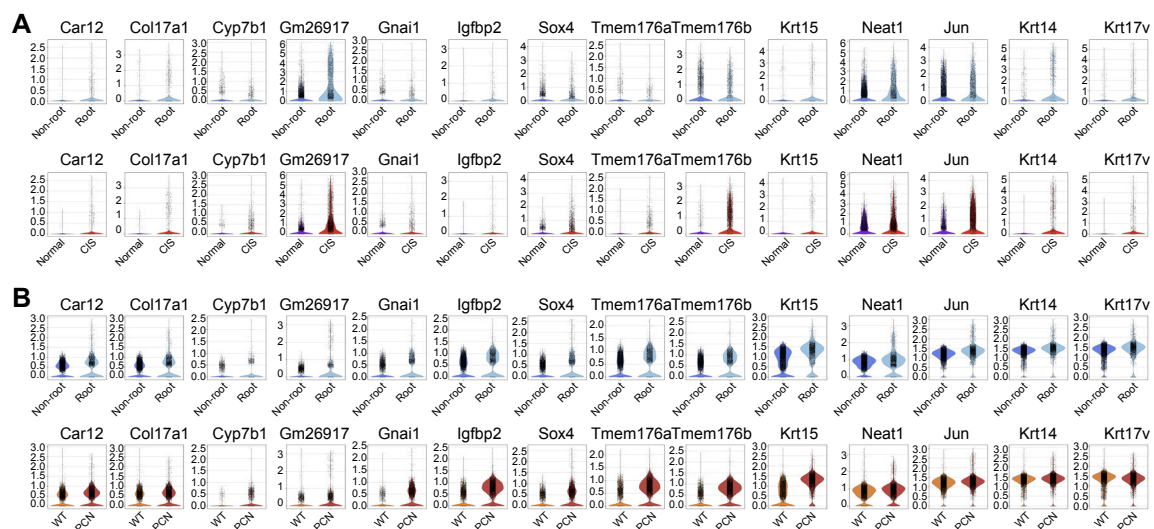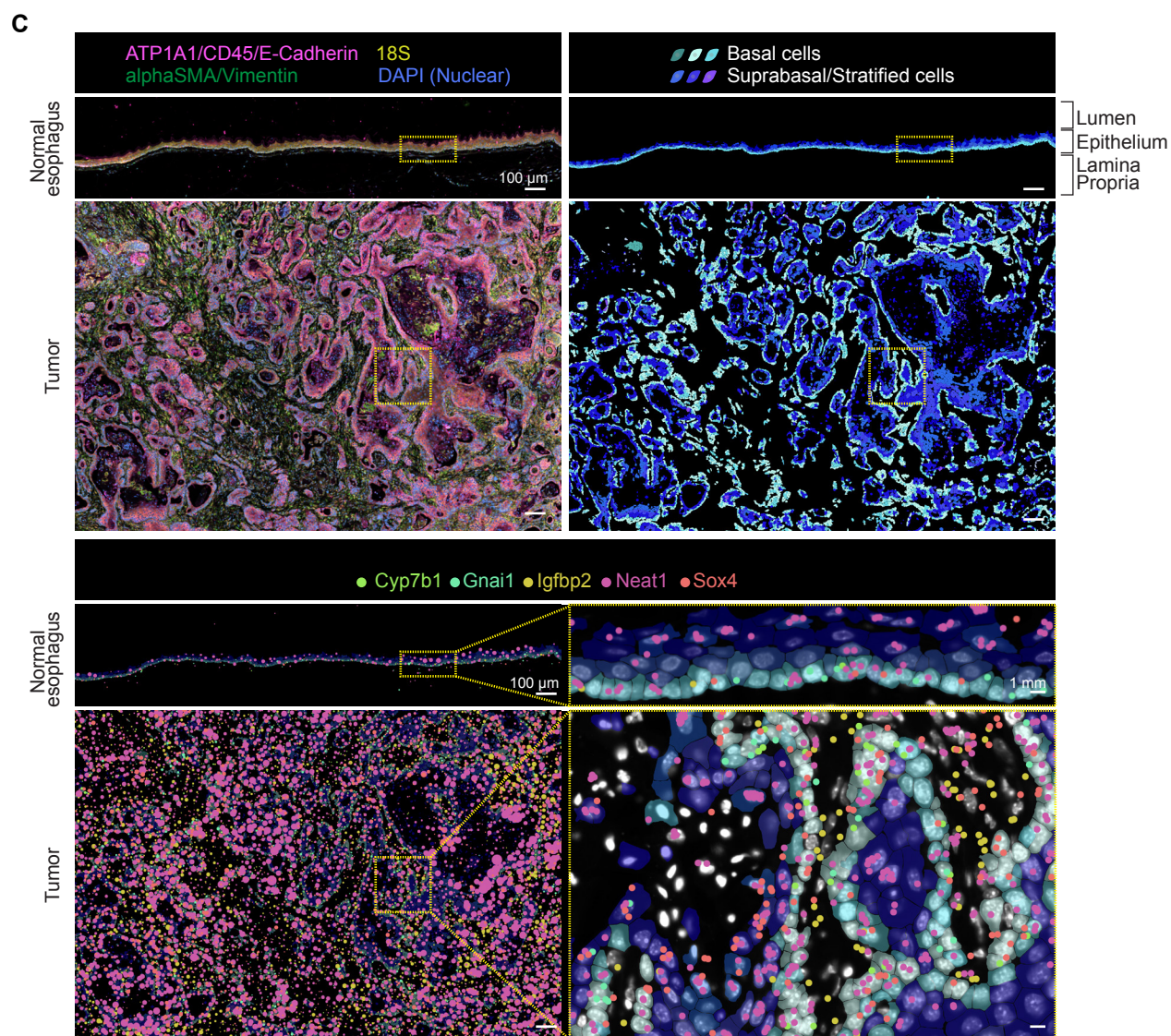

Supplement: Figure A1 — Identification of neoplastic root cell–enriched genes. (A) Differentially upregulated genes in root cell clusters from the integrated normal and cancer in situ (CIS) single-cell RNA sequencing (scRNA-seq) datasets. Violin plots compare expression between root versus nonroot cells, as well as normal versus CIS samples. (B)Differentially upregulated genes in root cell clusters from the integrated wild-type (WT) and PCN scRNA-seq datasets. Violin plots compare expression between root versus nonroot cells, as well as WT versus PCN samples. (C)Spatial transcriptomic validation (Xenium In Situ) of 5 genes consistently elevated in CIS and PCN root cells. Panels show tissue compartment annotation (top left), epithelial cell mapping (top right), and transcript localization at low (bottom left) and high (bottom right) magnification. [file mmc1.pdf]

**A**

Regulons specific to root cell clusters of Normal

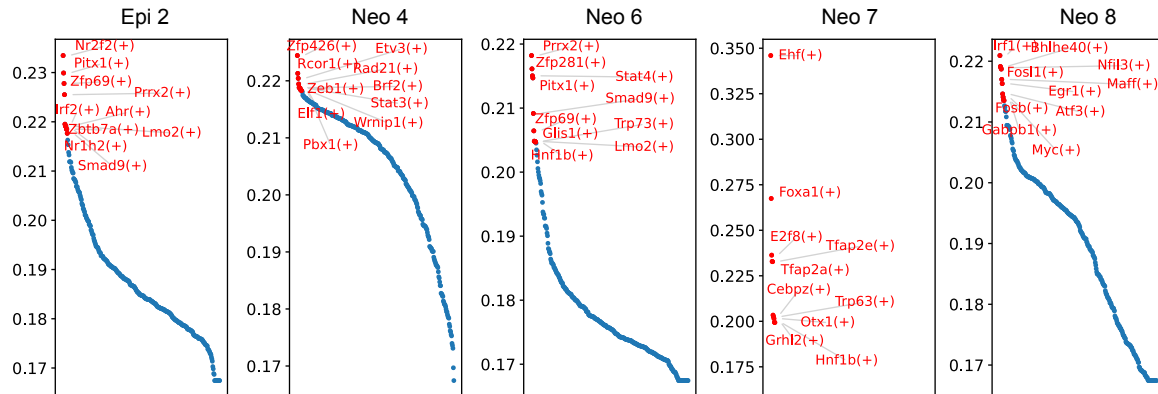

**B**

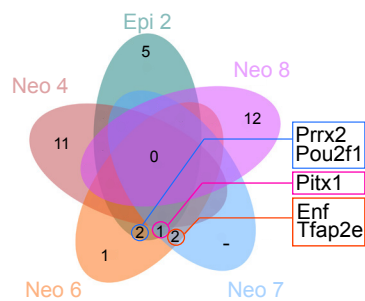

Supplement: Figure A2 — Gene regulatory network specific to cells-of-origin clusters of normal epithelial cells. (A) The gene regulatory network (GRN) analysis was performed on the cells-of-origin clusters from the normal dataset using the pySCENIC workflow. The representative top 10 regulons for each cluster are shown, ranked by their regulon activity scores. (B) The top 10 regulons from 5 rounds of analysis were compared to identify the shared regulons within each cluster. Regulons repeatedly detected across the 5 runs for each cluster were defined as consistently enriched and subsequently compared across the cells-of-origin clusters. [file mmc2.pdf]
