# Supplementary material for: Single-Cell Lineage Trajectory Defines Cyclin-Dependent Kinase Inhibitor–Sensitive Cells-of-Origin in Esophageal Squamous Cell Carcinoma
Source: Gastro Hep Adv. 2025 Dec 29;5(3):100874. doi: 10.1016/j.gastha.2025.100874 (PMC12865637; doi:10.1016/j.gastha.2025.100874)
Supplement: Extended PDF [file mmc3.pdf]

Gastro Hep Advances 2026;5:100874

ORIGINAL RESEARCH—BASIC

Single-Cell Lineage Trajectory Defines Cyclin-Dependent Kinase Inhibitor–Sensitive Cells-of-Origin in Esophageal Squamous Cell Carcinoma

Kyung-Pil Ko,[1] Jie Zhang,[1] Sohee Jun,[1] and Jae-Il Park[1,2,3]

[1]*Department of Experimental Radiation Oncology, Division of Radiation Oncology, The University of Texas MD Anderson Cancer Center, Houston, Texas;* [2]*Graduate School of Biomedical Sciences, The University of Texas MD Anderson Cancer Center, Houston, Texas; and* [3]*Program in Genetics and Epigenetics, The University of Texas MD Anderson Cancer Center, Houston, Texas*

BACKGROUND AND AIMS: Understanding the cells of origin is essential for overcoming therapy resistance and improving early intervention strategies in esophageal squamous cell carcinoma (ESCC). Despite recent advances in genomic profiling, the precise cellular hierarchies and molecular programs driving ESCC initiation remain poorly defined. **METHODS:** We utilized machine learning-based single-cell trajectory analysis on 4-nitroquinoline 1-oxide–induced murine models and genetically engineered organoids to identify cellular lineages during tumorigenesis. Combined with gene regulatory network analysis, we identified transcriptional drivers of tumor initiation and employed transcriptome-based drug repurposing to predict compounds targeting these initiating populations. **RESULTS:** Our analyses revealed multiple distinct epithelial clusters that function as cellular origins of ESCC, exhibiting diverse stem and progenitor signatures. Gene regulatory network analysis of these populations indicated activation of stem/progenitor cell regulators, including CEBPβ and TFAP2A/C. Translating these findings, a transcriptome-based drug repurposing screen identified 5 chemical candidates, 4 of which are potent cyclin-dependent kinase inhibitors, aligning with the frequent loss-of-function mutations in *TP53* and *CDKN2A* observed in ESCC. Notably, CDK inhibitors markedly inhibit ESCC cell proliferation. **CONCLUSION:** This research delineates the potential cellular origins of ESCC and their key regulons, thereby pioneering a single-cell-derived therapeutic strategy that exposes vulnerabilities in tumor-initiating cells.

Keywords: Esophageal Squamous Cell Carcinoma; Cancer Stem Cells; Single-Cell Transcriptomics; CDK Inhibitors

Introduction

Esophageal cancer is divided into esophageal squamous cell carcinoma (ESCC) and esophageal adenocarcinoma (EAC). ESCC accounts for over 80% of all cases of esophageal cancer and has a poor prognosis because of a lack of symptoms in the early stages.[1] The overall 5-year survival of patients with esophageal cancer ranges from 10% to 25%.[2] ESCC develops from squamous dysplasia as an invasive, typical histologic precursor lesion,[3] which impedes the early detection of the lesion and consequently results in late diagnosis, thereby adversely affecting patient survival.

Given that early diagnosis of ESCC may bring better outcomes,[2] understanding the genetic, cellular, and molecular mechanisms of esophageal neoplasia and ESCC initiation is imperative, which may improve the detection, diagnosis, treatment, and prevention of ESCC. However, the biology of ESCC initiation remains elusive.

The esophageal epithelium is composed of a proliferative basal layer and differentiated suprabasal layers of epithelial cells.[4] The basal epithelium of the murine esophagus contains both proliferating stem and transit-amplifying cells that self-renew and differentiate over the tissue's lifespan.[5] Three-dimensional organoids, which simulate physiological and pathological organ processes, have become a promising tool for studying stem cells and diseases.[6–8] We recently reformulated the culture media for esophageal organoids (EOs), which are cost-effective and superior to conventional ones, and established a new EO system that mimics ESCC's early lesion.[9,10] To understand the genetic mechanism of ESCC initiation, we genetically engineered EOs and identified the key genetic determinants (loss-of-function of *TP53*, *CDKN2A*, and *NOTCH1*) that initiate ESCC tumorigenesis and immune evasion.[11]

ESCC is mainly treated by surgery, while radiotherapy, chemotherapy, and chemoradiotherapy have limited efficacy.[12,13] Since cells-of-origin of cancer (also called cancer stem and progenitor cells) are likely responsible for therapy resistance, relapse, and metastasis,[14,15] targeting cells-of-origin has been highlighted to overcome the limitations

Abbreviations used in this paper: **4NQO, 4-nitroquinoline 1-oxide; CDK, cyclin-dependent kinase; CIS, cancer in situ; EO, esophageal organoid; ESCC, esophageal squamous cell carcinoma; PC, *Trp53 Cdkn2a* double knockout; PCN, *Trp53 Cdkn2a Notch1* triple knockout; PN, *Trp53 Notch1* double knockout; scRNA-seq, single-cell RNA sequencing; WT, wild-type.**

Most current article

Copyright © 2025 The Authors. Published by Elsevier Inc. on behalf of the AGA Institute. This is an open access article under the CC BY license (http://creativecommons.org/licenses/by/4.0/).
2772-5723
https://doi.org/10.1016/j.gastha.2025.100874

in cancer treatment.[16] To this end, many studies have focused on identifying specific biomarkers for such cellular origins.[17,18] Nonetheless, the current understanding of the cellular origin of cancer (especially in solid tumors) is still insufficient to pinpoint the specific cells for targeted treatment. This is partly due to the limitation of current approaches, which heavily depend on a single or a few biomarkers to define self-renewing tumor cells.

To overcome this, we employed single-cell transcriptomics and identified the earliest cell clusters serving as potential cellular origins of ESCC tumorigenesis. By leveraging genetically engineered organoids and single-cell transcriptomics, this study aimed to determine whether pharmacological interventions targeting cellular origins, as defined by single-cell transcriptomics, are sufficient to suppress ESCC tumorigenesis, laying a new foundation for developing ESCC therapies.

Methods

Cell Culture

Human ESCC cell lines, TE-1 and TE-12, were kindly provided by Dr Shumei Song. Cells were maintained in Roswell Park Memorial Institute 1640 supplemented with 10% fetal bovine serum at 37 C under 5% CO2 atmosphere. New vials of frozen stocks were routinely thawed and cultured for experiments, minimizing long-term passaging effects.

Colony Formation and Cell Viability Assays

For colony formation assays, 1×10^4 cells were plated in 60-mm dishes and cultured with medium refreshed every 2 days. Colonies were fixed with methanol for 20 minutes, rinsed with distilled water, and stained using 0.05% crystal violet. After 3 additional washes, plates were air-dried and imaged for quantification. For cell viability assays, 1×10^3 cells/well were seeded in 96-well plates. After 24 hours, the medium was replaced with drug-containing medium at varying concentrations, followed by 48 hours of incubation. Cell viability was assessed using the Cell Counting Kit-8 reagent (Dojindo) with a 4-hour incubation, and absorbance was measured at 450 nm (reference: 630 nm). Each condition was tested in triplicate. IC_{50} values were calculated using a four-parameter nonlinear logistic regression model in Graphpad Prism 10.

Preprocessing Single-Cell RNA Sequencing Data

Mouse 4-nitroquinoline 1-oxide (4NQO)–induced ESCC single-cell RNA sequencing (scRNA-seq) data were downloaded from the Genome Sequence Archive in BIG Data Center (Beijing Institute of Genomics, Chinese Academy of Sciences, http://gsa.big.ac.cn) under the accession number CRA002118.[19] Organoid datasets were generated in our prior study (accession number: GSE213929).[11] Data preprocessing was conducted using Scanpy (version 1.10.4).[20] Quality-control filters excluded cells expressing fewer than 100 genes and genes detected in fewer than three cells.

RNA Velocity–Based Lineage Trajectory Inference

RNA velocity, latent time, and partition-based graph abstraction-directed trajectories were analyzed using scVelo.[8] Briefly, spliced and unspliced Cell Ranger-generated matrices were merged, and genes with fewer than 20 counts were filtered. The top 2000 highly variable genes were selected. Data were size-normalized, log-transformed, and neighborhood moments computed. RNA velocities were estimated using the dynamical model and visualized on the uniform manifold approximation and projection embedding. For Dynamo analysis, the velocity vector field was constructed and characterized for topological features including curl, divergence, acceleration, and curvature using Dynamo package.[21]

Regulon Analysis and Visualization

Transcriptional regulatory network inference was performed using pySCENIC[22] to predict transcription factor–target interactions and evaluate regulon activity at the single-cell level. AUCell scores were computed per regulon and cell cluster to derive regulon specificity scores. To confirm consistency, analyses were repeated five times for each cluster, and the most prominent regulons were identified for visualization.

Gene Set Enrichment Analysis

Gene set enrichment analysis was carried out using the fgsea package[23] in R to identify significantly enriched biological processes. Genes from differential-expression analyses were ranked by scores obtained from Scanpy's rank_genes_groups function and input as preranked lists. msigdbr was used to access hallmark and curated gene sets (databases: Gene Ontology Biological Process and Kyoto Encyclopedia of Genes and Genomes).

Results

Identification of Multiple Cells-Of-Origin in the ESCC Models

To pinpoint the cells of origin responsible for ESCC tumorigenesis, we employed single-cell transcriptomics-based approaches. We analyzed scRNA-seq datasets derived from the esophageal tissues of mice treated with 4NQO, a potent carcinogenic agent.[19] This 4NQO-treated mouse model exhibits a progressive series of lesions that faithfully mimic the developmental stages of ESCC tumorigenesis, encompassing inflammation, hyperplasia, dysplasia, and cancer in situ (CIS) (Figure 1A). Notably, our recent studies have demonstrated a high degree of similarity between the single-cell transcriptomes of 4NQO-treated ESCC mouse models and those of human ESCC.[11,24] Transcriptomes of cells from five different stages were integrated and annotated for isolating epithelial cells from other cell types, such as fibroblasts (Figure 1B). Cell clusters that are dominant in the normal dataset were annotated as normal epithelial cells (Epi 1–8), while CIS-abundant cell clusters were named as neoplastic cells (Neo 1–11).

Next, we harnessed the power of cell lineage trajectory inference analysis, a cutting-edge method, on the scRNA-seq datasets sourced from 4NQO ESCC mouse models. Employing 3 distinct analytical packages, namely RNA velocity (scVelo; based on RNA splicing), CytoTRACE (based on RNA content), and Dynamo (utilizing machine

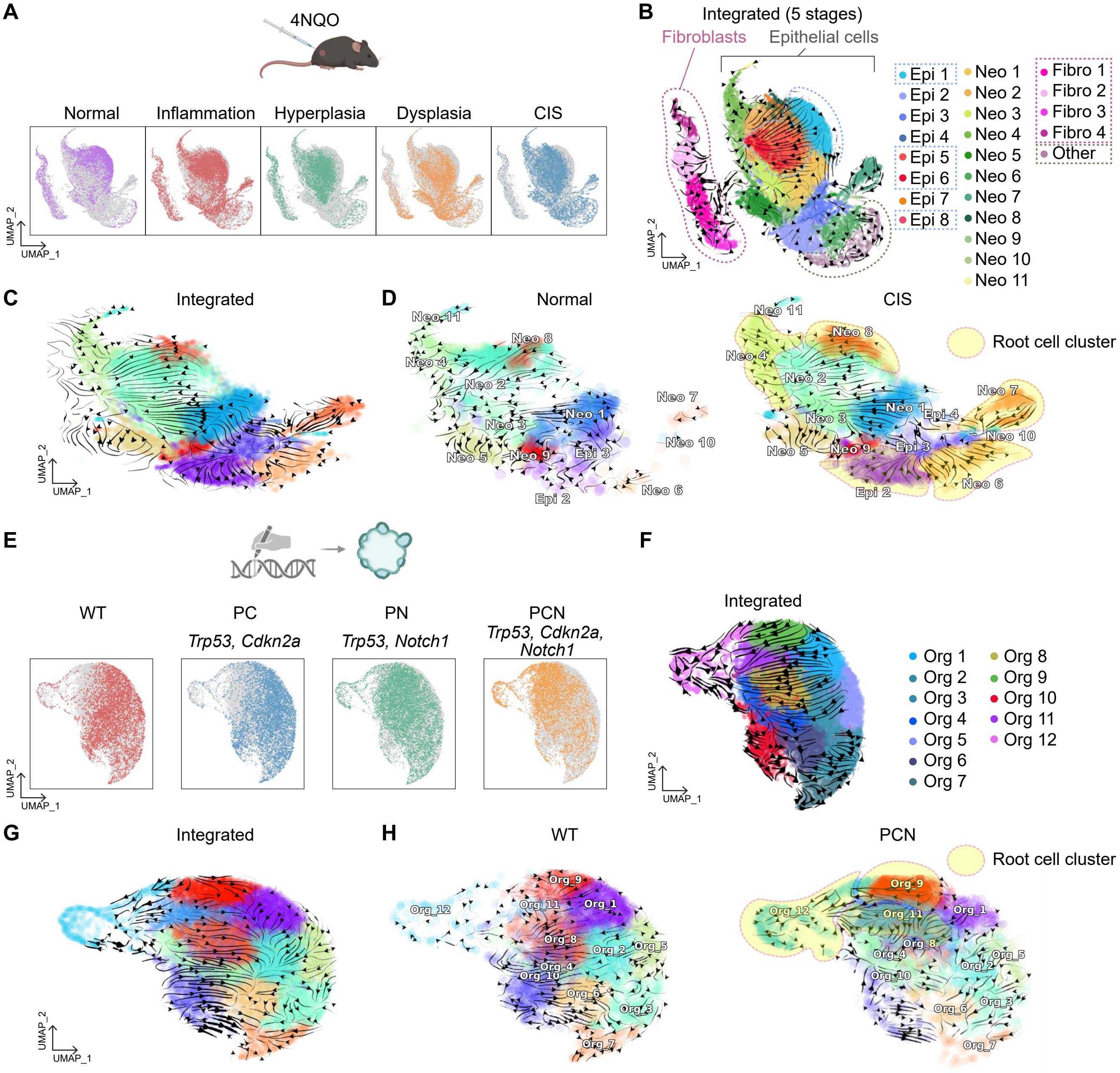

Figure 1. Identification of cells-of-origin clusters of ESCC. (A) UMAPs from the integrated dataset from 4NQO-treated mouse esophageal samples. CD45(−) cells from Normal, Inflammation, Hyperplasia, Dysplasia, and CIS datasets were integrated using the Harmony package. (B) RNA velocity-based UMAP from the integrated dataset from 4NQO-treated mouse esophageal samples (normal, inflammation, hyperplasia, dysplasia, and CIS). RNA velocity was calculated with the scVelo package. Proliferating cells or differentiated cells were annotated with Epi 1–8, and the fibroblasts were annotated with Fibro 1–4 based on the marker gene expression. Cell clusters that have mixed gene expression patterns of proliferating cells and stratified cells were annotated with Neo 1-11, and a cluster that does not express epithelial cell markers was named Other. (C) Normal cell lineage-related (Epi 1, 5–8), Fibroblast (Fibro 1–4), and Other clusters were excluded, and the RNA velocity was calculated again using the Dynamo package. (D) Normal and CIS datasets were separated from the integrated data, and the cell differentiation streams were displayed using Dynamo. Cells-of-origin clusters (Epi 2, Neo 4, 6, 7, and 8) specific to CIS were highlighted. (E) The integrated dataset was segregated with each genotype (PC, PN, and PCN) and shown on the UMAP. (F) The UMAP, showing the integrated organoid datasets (WT, PC, PN, and PCN), and RNA velocity calculated by scVelo, was displayed on the UMAP. (G) RNA velocity was recalculated with Dynamo. (H) RNA velocity of WT and PCN subsets from the integrated dataset was displayed separately. Cells-of-origin clusters (Org_9, 11, and 12) enriched in PCN were highlighted.

learning),[21,25,26] we visualized cell lineage trajectories (Figure 1B). To directly compare the change of cellular trajectories in normal and CIS esophagi, we isolated only normal and CIS datasets (Figure 1C). In contrast to the cells of origin identified in normal esophagi, the CIS stage displayed 5 distinctive cells of origin clusters, Neo 4, 6, 7, 8, and epi 2 (Figure 1C and D).

Moreover, we extended our analysis to include scRNA-seq datasets from genetically engineered EOs that we recently pioneered.[11] These EOs encompass normal EOs (wild-type [WT]), *Trp53 Cdkn2a* knockout (PC), *Trp53 Notch1* knockout (PN), and *Trp53 Cdkn2a Notch1* triple knockout (PCN) EOs, with the latter demonstrating the development of invasive ESCC tumors[11] (Figure 1E).

We integrated 4 different organoid datasets and annotated each epithelial cell cluster (Org 1–12) (Figure 1F). In this integrated dataset, 2 cell clusters, Org 2 and Org 9, were identified as the major cells of origin. To exclude masking effect from PC and PN data, we isolated only WT and PCN datasets and compared their cell lineages (Figure 1G). Intriguingly, the WT organoid exhibited an opposite cellular lineage direction compared to the previous integrated dataset. The Org 4 cell cluster was the cell-of-origin of normal EOs and differentiates into others (Figure 1H). Remarkably, 3 cell clusters, Org 9, 11, and 12, were revealed as initiating cell clusters of PCN EOs, confirming multiple cellular origins in the neoplastic cells (Figure 1H). The lineage directions of PCN were similar to those of the previous integrated dataset, likely reflecting the transcriptomic proximity of PC and PN to the PCN data. These results indicated that ESCC cells are heterogeneous, likely driven by various cells of origin.

We also identified a set of genes enriched specifically in the root clusters of both CIS and PCN datasets. Differential expression analyses comparing root versus nonroot cells yielded 15 genes consistently elevated in neoplastic root clusters relative to Normal and WT (Figure A1A and B). Expression of these genes was similarly higher in CIS and PCN bulk populations, supporting their specificity to neoplastic root cells rather than normal epithelial cells. To determine the spatial distribution of these markers, we examined Xenium In Situ spatial transcriptomic data from normal esophagus and tumor tissues. Of the 14 genes detectable in the Xenium platform, 5 were present in the gene panel (Figure A1C).[24] Consistent with the single-cell data, these markers were enriched in tumor regions compared to normal epithelium. In normal tissue, Igfbp2, Sox4, Cyp7b1, and Gnai1 were localized predominantly to the basal layer, whereas Neat1 was expressed across basal and suprabasal layers. This pattern was similarly observed in the tumor sample although tumor tissues are more heterogenous than normal esophagus. These results suggested that neoplastic root cells share basal-like epithelial characteristics.

Gene Regulatory Networks of Cells of Origin

Next, we analyzed the gene regulatory networks characterizing each cell-of-origin cluster within the CIS scRNA-seq dataset by using the pySCENIC package.[27] Across 5 independent pySCENIC runs, we identified the top 10 regulons for each cells-of-origin cluster (Epi2, Neo4, 6-8) and determined those that were consistently enriched. From this analysis, 8 regulons (Prrx2, Cebpb, Zbtb7b, Snai2, Tfap2a, Tfap2c, Trp63, and Snai3) were consistently activated in the cells-of-origin clusters of CIS (Figure 2A and B). PRRX2 serves as a marker

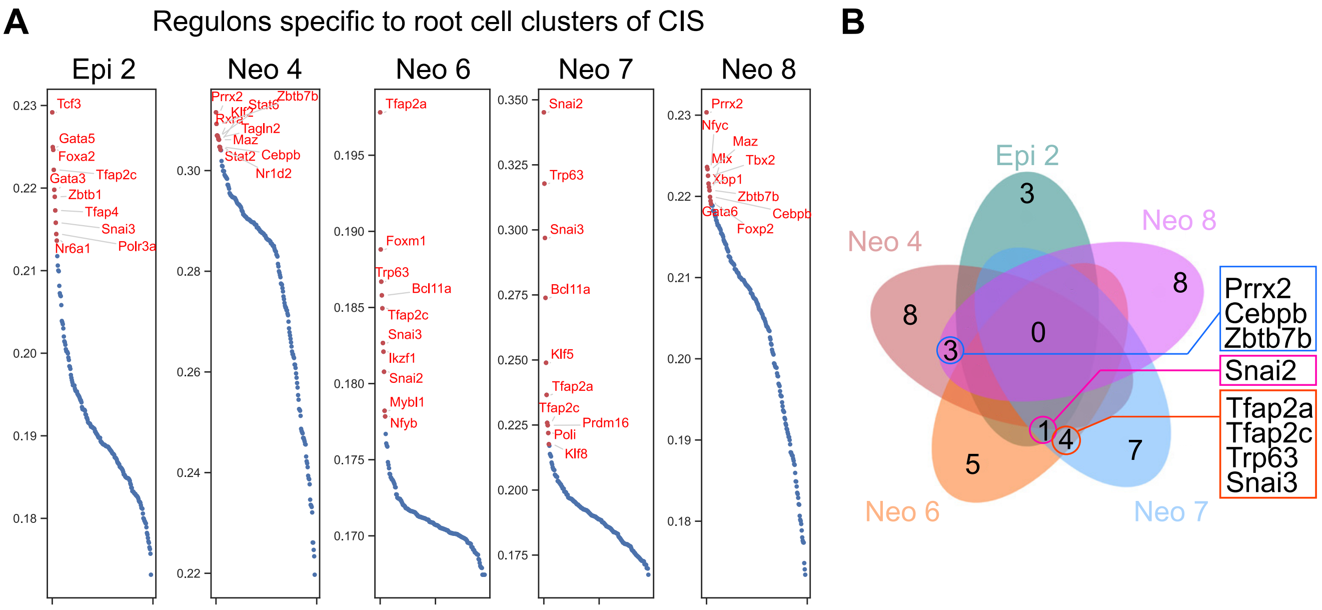

Figure 2. GRN specific to cells-of-origin clusters of ESCC. (A) The GRN was calculated from the cells-of-origin clusters of the CIS dataset. The pySCENIC package was used for regulon analysis, and the top 10 regulons for each cluster were displayed based on their regulon scores. Each cluster was calculated 5 times with pySCENIC. (B) The top 10 regulons from 5 rounds of analysis were compared to identify the shared regulons within each cluster. Only shared regulons from 5 rounds of each cluster were compared with those of other cells-of-origin clusters, and eight regulons (Prrx2, Cebpb, Zbtb7b, Snai2, Tfap2a, Tfap2c, Trp63, and Snai3) were identified to overlap in at least 2 clusters. GRN, gene regulatory network.

for pituitary stem/progenitor cells,[28,29] while CEBPβ plays a regulatory role in hematopoietic and skeletal stem cells.[30,31] SNAI2 is known for its control over epidermal progenitor cells,[32] and TFAP2A/C is associated with the regulation of pluripotent stem cell differentiation.[33] Moreover, p63/TP63 is recognized for its role in modulating the proliferation of epithelial stem and progenitor cells.[34]

To evaluate whether these regulons are specifically associated with neoplastic root cells, we performed an additional pySCENIC analysis on the normal dataset and compared the resulting regulon profiles with those from CIS (Figure A2A). Among the 8 regulons shared across CIS root cells, only Prrx2 was also detected in the normal dataset. This limited overlap demonstrates that normal epithelial cells and neoplastic cells engage fundamentally distinct gene regulatory programs. (Figure A2B).

Signaling Pathways in the ESCC Initiating Cell Clusters

Since a significant number of epithelial cells from the normal esophagus were also observed in the CIS-enriched initiating cell clusters, such as Neo 4 and Neo 8, we compared the signaling pathways between the normal and CIS datasets within these cell clusters. In the Neo 4 cell cluster, cytokine-mediated pathways, including interleukin-17 and tumor necrosis factor signaling, were enriched in the CIS compared to normal esophagus, indicating more active cell–cell interactions in CIS than normal cells (Figure 3A). In the Neo 8 cluster, mitochondrial electron transport and oxidative phosphorylation pathways were stronger in CIS than in normal cells, suggesting enhanced energy production through oxidative phosphorylation metabolism. These results suggest that ESCC-initiating cells are heterogeneous, with distinct intercellular and intracellular processes.

Identification of Drug Candidates Potentially Targeting Cells-Of-Origin of ESCC

Having identified heterogeneous cellular processes in the initiating cell clusters through scRNA-seq analysis (Figure 3), our subsequent objective was to pinpoint chemicals or drugs that specifically, yet integratively, target these cell-of-origin clusters. To achieve this, we employed a transcriptome-based drug repurposing algorithm, as recently performed.[35,36] In a manner akin to the Connectivity Map,[37] we conducted a data-driven analysis focusing on drug mode-of-action and drug repositioning. Rather than considering the entire transcriptome, we generated lists of differentially expressed genes that were unique to each of the cells-of-origin clusters. Subsequently, we utilized the L1000CDS[2] tool[38] and identified 5 promising chemical candidates (CGP60474, Flavopiridol, AZD-5438, SNS-032, and daunorubicin) with their potential to target the cells-of-origin clusters (Figure 4A). Of note, CGP60474 is an inhibitor of cyclin-dependent kinases (CDK) and protein kinase C. Flavopiridol, also known as alvocidib, holds Food and Drug Administration approval as a CDK inhibitor. AZD-5438 similarly acts as a CDK inhibitor, blocking CDK1, CDK2, and CDK9. Meanwhile, SNS-032 is

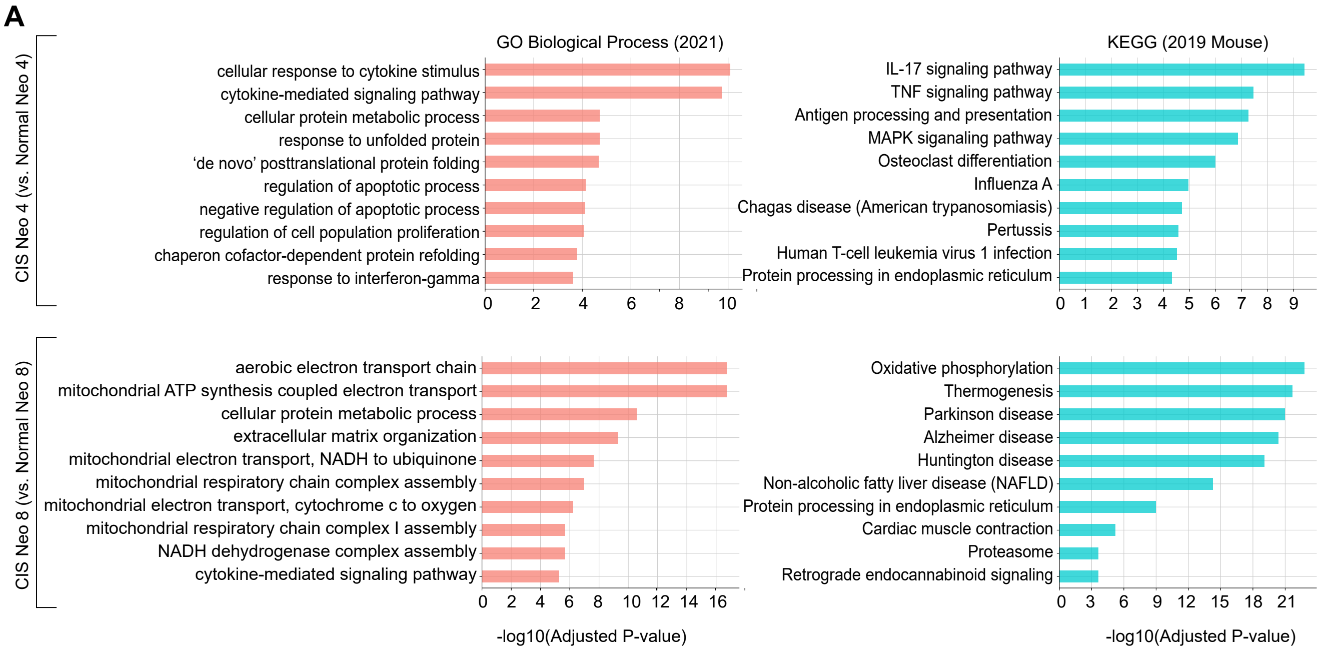

Figure 3. GSEA analysis of initiating cells between normal and CIS. (A) GSEA from differentially expressed genes between CIS and Normal cells within the Neo 4 and Neo 8 clusters. The top 10 significant pathways from GO Biological Process and KEGG databases are shown. ATP, adenosine triphosphate; GO, gene ontology; GSEA, gene set enrichment analysis; KEGG, kygoto encyclopedia of gene and genomes; MAPK, mitogen-activated protein kinase; NADH, nicotinamide adenine dinucleotide hydride.

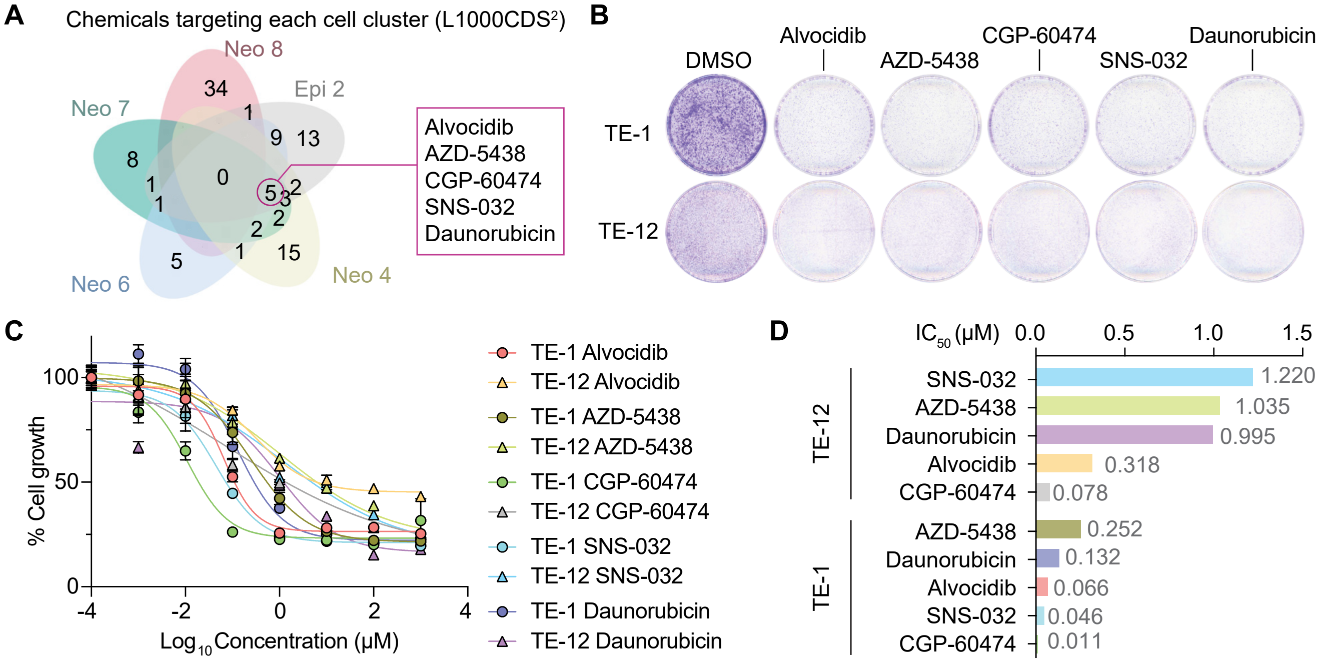

Figure 4. Identification of chemicals targeting cells-of-origin of ESCC. (A) Drug candidates were identified from L1000CDS[2] analysis. DEGs from CIS and normal in each cell-of-origin cluster were used for analysis, and 5 chemicals were shared in four clusters. B. Colony formation assay showing the growth inhibitory effect of 5 drugs in TE-1 and TE-12 human ESCC cell lines. Cells were stained with Crystal violet 48 hours after chemical/drug treatment (10 μM). Representative images are shown (n = 3). C. The impact of 5 drugs/chemicals (alvocidib, AZD-5438, CGP-60474, SNS-032, and daunorubicin) on the cell growth of TE-1 and TE-12 cells was analyzed using the CCK-8 assay. Different doses (10^{-4} μM–10^{3} μM) were tested after 48 h of treatment. DMSO (vehicle) served as control. D. IC_{50} values of the candidate drugs are shown. DEGs, differentially expressed genes; DMSO, dimethyl sulfoxide.

recognized for its inhibition of CDK2, CDK7, and CDK9. Daunorubicin, an FDA-approved agent, functions as a DNA intercalating agent akin to doxorubicin, primarily used in the treatment of leukemia. Among these five chemicals, CGP-60474 (with an IC_{50} of 0.011 and 0.078 mM) and Flavopiridol (with an IC_{50} of 0.066 and 0.318 mM) exhibited noteworthy growth inhibitory effects on TE-1 and TE-12 human ESCC cell lines (Figure 4B–D).

Discussion

Understanding the cells of origin has mainly been emphasized in developmental biology and regenerative medicine. Starting from long-term labeled cell detection, by using genetically engineered mouse models, genetic labeling-based cell lineage tracking and cell ablation approaches have revealed several tissue stem and progenitor cells.[39–45]

The concept of targeting cells of origin, especially cancer stem cells, has emerged as a promising strategy to combat therapy resistance, reduce relapse rates, and impede metastasis in various malignancies.[14,15,46–48] Nevertheless, the current understanding of cancer cell origins, particularly in solid tumors, remains incomplete, hindering the precise identification and targeting of these critical cellular populations. This limitation partly stems from the conventional methodologies, which predominantly rely on a single or a limited set of biomarkers for the identification and characterization of self-renewing tumor cells. The recent advent of single-cell transcriptomics and genomics has enabled a comprehensive understanding of cellular classifications, lineage specification, and plasticity in development, tissue regeneration, and tumorigenesis.[9,11,21,24–26,36,49–53]

To identify the cells of origin in ESCC, we analyzed scRNA-seq datasets of 4NQO ESCC mouse models[19] and genetically engineered organoids. A 4NQO ESCC mouse model recapitulates ESCC tumorigenesis, including inflammation, hyperplasia, dysplasia, and CIS, as analyzed by scRNA-seq datasets.[11] By using scVelo, CytoTRACE, and Dynamo, we identified the distinct cell lineage trajectories of CIS compared to those of normal esophagus. Machine learning-based Dynamo best identified the 5 cell clusters (Neo 2, 4, 6, 8, and epi 2) serving as cells-of-origin in CIS (Figure 1). Further analyses identified the key gene regulatory networks specifically activated in the cells-of-origin of CIS compared to the normal esophagus (Figures 2 and 3). Notably, we identified five drug or chemical candidates (CGP60474, Flavopiridol, AZD-5438, SNS-032, and daunorubicin) that significantly inhibited tumor cell growth (Figure 4).

Interestingly, 4 of these chemical candidates (CGP60474, Flavopiridol, AZD-5438, and SNS-032) were known to be potent and selective inhibitors of CDKs, implying therapeutic vulnerabilities in cyclin-dependent

cell cycle regulation. This might be originated from clonal evolution driven by consensus genetic alterations during ESCC development. Unlike other types of cancer, ESCC patients show an extremely high frequency of *TP53* mutation and loss of *CDKN2A* genes, which are more than 90% and 70%, respectively.[11,54–56] Considering their overlapping roles in RB1-mediated cell cycle control, better therapeutic outcomes for ESCC can be achieved by targeting cyclin-dependent cell cycle. Consistent with our findings, more than 62% of ESCC cases exhibited genetic profiles of disrupted G1/S transition control.[57]

There were several preclinical trials to suppress ESCC growth using CDK inhibitors alone or combinatorial therapy.[58–60] ESCC cell proliferation was significantly reduced by dalpiciclib and SNS-032, and sensitivity to radiotherapy or chemotherapy was enhanced by CDK inhibitors, such as palbociclib. Although CDK inhibitors show promising results in ESCC cell lines, further research is necessary to determine whether their tumor suppressive effects stem from blocking cancer cell stemness or from cytostatic mechanisms, using patient-derived xenografts or organoids.

Conclusion

This study elucidates the potential cellular origins and driving regulons involved in ESCC tumorigenesis. Notably, we have translated these findings into a therapeutic strategy by pioneering the application of single-cell transcriptome data to identify compounds that could suppress cancer cell stemness, thereby potentially targeting the cells-of-origin of tumor heterogeneity, metastatic progression, and therapeutic resistance. This study provides a new foundation for advancing the understanding and treatment of ESCC.

Supplementary Materials

Material associated with this article can be found, in the online version, at https://doi.org/10.1016/j.gastha.2025.100874.

References

1. Glenn TF. Esophageal cancer. Facts, figures, and screening. Gastroenterol Nurs 2001;24:271–273, quiz 274-275.
2. Pennathur A, Gibson MK, Jobe BA, et al. Oesophageal carcinoma. Lancet 2013;381:400–412.
3. Wang GQ, Abnet CC, Shen Q, et al. Histological precursors of oesophageal squamous cell carcinoma: results from a 13 year prospective follow up study in a high risk population. Gut 2005;54:187–192.
4. Whelan KA, Muir AB, Nakagawa H. Esophageal 3D culture systems as modeling tools in esophageal epithelial pathobiology and personalized medicine. Cell Mol Gastroenterol Hepatol 2018;5:461–478.
5. DeWard AD, Cramer J, Lagasse E. Cellular heterogeneity in the mouse esophagus implicates the presence of a nonquiescent epithelial stem cell population. Cell Rep 2014;9:701–711.
6. Tuveson D, Clevers H. Cancer modeling meets human organoid technology. Science 2019;364:952–955.
7. Li X, Francies HE, Secrier M, et al. Organoid cultures recapitulate esophageal adenocarcinoma heterogeneity providing a model for clonality studies and precision therapeutics. Nat Commun 2018;9:2983.
8. La Manno G, Soldatov R, Zeisel A, et al. RNA velocity of single cells. Nature 2018;560:494–498.
9. Ko KP, Zhang J, Park JI. Establishing transgenic murine esophageal organoids. STAR Protoc 2022;3:101317.
10. Zheng B, Ko KP, Fang X, et al. A new murine esophageal organoid culture method and organoid-based model of esophageal squamous cell neoplasia. iScience 2021;24:103440.
11. Ko KP, Huang Y, Zhang S, et al. Key genetic determinants driving esophageal squamous cell carcinoma initiation and immune evasion. Gastroenterology 2023;165:613–628.e20.
12. Muro K, Hamaguchi T, Ohtsu A, et al. A phase II study of single-agent docetaxel in patients with metastatic esophageal cancer. Ann Oncol 2004;15:955–959.
13. Kato K, Tahara M, Hironaka S, et al. A phase II study of paclitaxel by weekly 1-h infusion for advanced or recurrent esophageal cancer in patients who had previously received platinum-based chemotherapy. Cancer Chemother Pharmacol 2011;67:1265–1272.
14. Dean M, Fojo T, Bates S. Tumour stem cells and drug resistance. Nat Rev Cancer 2005;5:275–284.
15. Hermann PC, Huber SL, Herrler T, et al. Distinct populations of cancer stem cells determine tumor growth and metastatic activity in human pancreatic cancer. Cell Stem Cell 2007;1:313–323.
16. Clevers H. The cancer stem cell: premises, promises and challenges. Nat Med 2011;17:313–319.
17. Yan Y, Zuo X, Wei D. Concise review: emerging role of CD44 in cancer stem cells: a promising biomarker and therapeutic target. Stem Cells Transl Med 2015;4:1033–1043.
18. Walcher L, Kistenmacher AK, Suo H, et al. Cancer stem cells-origins and biomarkers: perspectives for targeted personalized therapies. Front Immunol 2020;11:1280.
19. Yao J, Cui Q, Fan W, et al. Single-cell transcriptomic analysis in a mouse model deciphers cell transition states in the multistep development of esophageal cancer. Nat Commun 2020;11:3715.
20. Wolf FA, Angerer P, Theis FJ. SCANPY: large-scale single-cell gene expression data analysis. Genome Biol 2018;19:15.
21. Qiu X, Zhang Y, Martin-Rufino JD, et al. Mapping transcriptomic vector fields of single cells. Cell 2022;185:690–711.e45.
22. Aibar S, Gonzalez-Blas CB, Moerman T, et al. SCENIC: single-cell regulatory network inference and clustering. Nat Methods 2017;14:1083–1086.
23. Korotkevich G, Sukhov V, Budin N, et al. Fast gene set enrichment analysis. bioRxiv 2021;060012.
24. Jang J, Ko KP, Zhang J, et al. Deciphering Precursor cell dynamics in esophageal preneoplasia via genetic barcoding and single-cell transcriptomics. Proc Natl Acad Sci U S A 2025;122:e2509534122.
25. Bergen V, Lange M, Peidli S, et al. Generalizing RNA velocity to transient cell states through dynamical modeling. Nat Biotechnol 2020;38:1408–1414.

26. Gulati GS, Sikandar SS, Wesche DJ, et al. Single-cell transcriptional diversity is a hallmark of developmental potential. Science 2020;367:405–411.
27. Van de Sande B, Flerin C, Davie K, et al. A scalable SCENIC workflow for single-cell gene regulatory network analysis. Nat Protoc 2020;15:2247–2276.
28. Higuchi M, Kato T, Yoshida S, et al. PRRX1- and PRRX2-positive mesenchymal stem/progenitor cells are involved in vasculogenesis during rat embryonic pituitary development. Cell Tissue Res 2015;361:557–565.
29. Shintani A, Higuchi M. Isolation of PRRX1-positive adult pituitary stem/progenitor cells from the marginal cell layer of the mouse anterior lobe. Stem Cell Res 2021;52:102223.
30. de Laval B, Maurizio J, Kandalla PK, et al. C/EBPbeta-dependent epigenetic memory induces trained immunity in hematopoietic stem cells. Cell Stem Cell 2020;26:657–674.e58.
31. Wang J, Yang C, Kong F, et al. Cebpb regulates skeletal stem cell osteogenic differentiation and fracture healing via the WNT/beta-catenin pathway. Stem Cells Int 2022;2022:2091615.
32. Mistry DS, Chen Y, Wang Y, et al. SNAI2 controls the undifferentiated state of human epidermal progenitor cells. Stem Cells 2014;32:3209–3218.
33. Krendl C, Shaposhnikov D, Rishko V, et al. GATA2/3-TFAP2A/C transcription factor network couples human pluripotent stem cell differentiation to trophectoderm with repression of pluripotency. Proc Natl Acad Sci U S A 2017;114:E9579–E9588.
34. Senoo M, Pinto F, Crum CP, et al. p63 is essential for the proliferative potential of stem cells in stratified epithelia. Cell 2007;129:523–536.
35. Kim MJ, Cervantes C, Jung YS, et al. PAF remodels the DREAM complex to bypass cell quiescence and promote lung tumorigenesis. Mol Cell 2021;81:1698–1714.e6.
36. Kim B, Huang Y, Ko KP, et al. PCLAF-DREAM drives alveolar cell plasticity for lung regeneration. Nat Commun 2024;15:9169.
37. Lamb J, Crawford ED, Peck D, et al. The connectivity map: using gene-expression signatures to connect small molecules, genes, and disease. Science 2006;313:1929–1935.
38. Duan Q, Reid SP, Clark NR, et al. L1000CDS(2): LINCS L1000 characteristic direction signatures search engine. NPJ Syst Biol Appl 2016;2:16015.
39. Suh HN, Kim MJ, Jung YS, et al. Quiescence exit of Tert (+) stem cells by Wnt/beta-catenin is indispensable for intestinal regeneration. Cell Rep 2017;21:2571–2584.
40. Kim MJ, Xia B, Suh HN, et al. PAF-Myc-controlled cell stemness is required for intestinal regeneration and tumorigenesis. Dev Cell 2018;44:582–596.e4.
41. Suh HN, Kim MJ, Lee SH, et al. Yap/Taz-activated tert-expressing acinar cells are required for pancreatic regeneration. bioRxiv 2021.
42. VanHorn S, Morris SA. Next-generation lineage tracing and fate mapping to interrogate development. Dev Cell 2021;56:7–21.
43. Yao M, Ren T, Pan Y, et al. A new generation of lineage tracing dynamically records cell fate choices. Int J Mol Sci 2022;23:5021.
44. Serrano A, Berthelet J, Naik SH, et al. Mastering the use of cellular barcoding to explore cancer heterogeneity. Nat Rev Cancer 2022;22:609–624.
45. Kretzschmar K, Watt FM. Lineage tracing. Cell 2012;148:33–45.
46. Clara JA, Monge C, Yang Y, et al. Targeting signalling pathways and the immune microenvironment of cancer stem cells - a clinical update. Nat Rev Clin Oncol 2020;17:204–232.
47. Clarke MF. Clinical and therapeutic implications of cancer stem cells. N Engl J Med 2019;380:2237–2245.
48. Gupta PB, Pastushenko I, Skibinski A, et al. Phenotypic plasticity: driver of cancer initiation, progression, and therapy resistance. Cell Stem Cell 2019;24:65–78.
49. Seo Y, Zhang S, Jang J, et al. Actin dysregulation induces neuroendocrine plasticity and immune evasion: a vulnerability of small cell lung cancer. Nat Commun 2025;17:386.
50. Kim B, Zhang S, Huang Y, et al. CRACD loss induces neuroendocrine cell plasticity of lung adenocarcinoma. Cell Rep 2024;43:114286.
51. Ko KP, Zhang S, Huang Y, et al. Tumor niche network-defined subtypes predict immunotherapy response of esophageal squamous cell cancer. iScience 2024;27:109795.
52. Zou G, Huang Y, Zhang S, et al. E-cadherin loss drives diffuse-type gastric tumorigenesis via EZH2-mediated reprogramming. J Exp Med 2024;221:e20230561.
53. Dhar SS, Ko KP, Jang J, et al. KMT2D temporally activates neuronal transcriptional factor genes to mediate cerebellar granule cell differentiation. Sci Adv 2025;11:eadu7174.
54. Wu Z, Zhou J, Zhang X, et al. Reprogramming of the esophageal squamous carcinoma epigenome by SOX2 promotes ADAR1 dependence. Nat Genet 2021;53:881–894.
55. Sawada G, Niida A, Uchi R, et al. Genomic landscape of esophageal squamous cell carcinoma in a Japanese population. Gastroenterology 2016;150:1171–1182.
56. Gao YB, Chen ZL, Li JG, et al. Genetic landscape of esophageal squamous cell carcinoma. Nat Genet 2014;46:1097–1102.
57. Song Y, Li L, Ou Y, et al. Identification of genomic alterations in oesophageal squamous cell cancer. Nature 2014;509:91–95.
58. Qin WJ, Su YG, Ding XL, et al. CDK4/6 inhibitor enhances the radiosensitization of Esophageal Squamous Cell Carcinoma (ESCC) by activating autophagy signaling via the suppression of mTOR. Am J Transl Res 2022;14:1616–1627.
59. Zeng H, Yang H, Song Y, et al. Transcriptional inhibition by CDK7/9 inhibitor SNS-032 suppresses tumor growth and metastasis in esophageal squamous cell carcinoma. Cell Death Dis 2021;12:1048.
60. Wang J, Li Q, Yuan J, et al. CDK4/6 inhibitor-SHR6390 exerts potent antitumor activity in esophageal squamous cell carcinoma by inhibiting phosphorylated Rb and inducing G1 cell cycle arrest. J Transl Med 2017;15:127.

Received December 1, 2025. Accepted December 22, 2025.

Correspondence:
Address correspondence to: Kyung-Pil Ko, PhD, Division of Radiation Oncology, Department of Experimental Radiation Oncology, The University of Texas MD Anderson Cancer Center, Houston, Texas. e-mail: kko1@mdanderson.org; or Jae-Il Park, PhD, Division of Radiation Oncology,

Department of Experimental Radiation Oncology, The University of Texas MD Anderson Cancer Center, Houston, Texas. e-mail: jaeil@mdanderson.org.

Acknowledgments:
The authors thank Dr Shumei Song for providing them with the TE-1 and TE-12 cell lines.

Authors' Contributions:
Kyung-Pil Ko: Conceptualization, methodology, investigation, software, analysis, data curation, writing, visualization, funding acquisition. Jie Zhang: Investigation. Sohee Jun: Investigation. Sohee Jun: Conceptualization, analysis, writing, visualization, supervision, project administration, funding acquisition.

Conflicts of Interest:
The authors disclose no conflicts.

Funding:
This work was supported by grants from the National Cancer Institute (CA286761 to K.-P.K. and CA278971 to J.-I.P.).

Ethical Statement:
Institutional review board approval was not required, as this study analyzed publicly available datasets generated in previously published studies.

Data Transparency Statement:
Public Xenium In Situ data were downloaded from the GEO database under accession number GSE289049. The code used to reproduce the analyses described in this manuscript can be accessed via GitHub (https://github.com/jaeilparklab/ESCC_CDKi).

Reporting Guidelines:
Not applicable.

Gastro Hep Advances, Volume 5

Supplemental information

Single-Cell Lineage Trajectory Defines Cyclin-Dependent Kinase Inhibitor–Sensitive Cells-of-Origin in Esophageal Squamous Cell Carcinoma

Kyung-Pil Ko, Jie Zhang, Sohee Jun, and Jae-Il Park

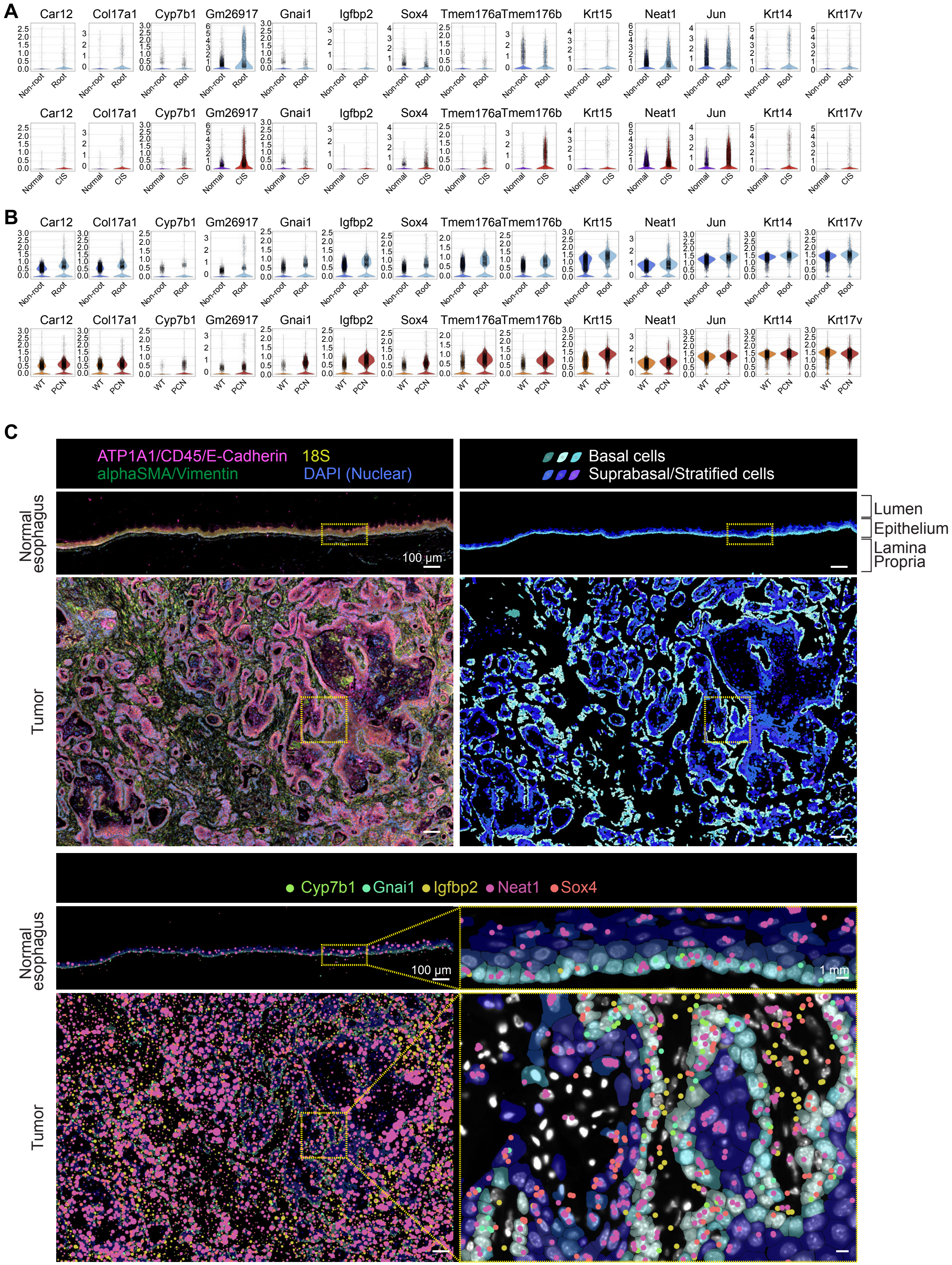

A
B
C
Car12
Col17a1
Cyp7b1
Gm26917
Gnai1
Igfbp2
Sox4
Tmem176a
Tmem176b
Krt15
Neat1
Jun
Krt14
Krt17v
Non-root
Root
Normal
CIS
WT
PCN
ATP1A1/CD45/E-Cadherin
18S
alphaSMA/Vimentin
DAPI (Nuclear)
Basal cells
Suprabasal/Stratified cells
Normal esophagus
Tumor
Lumen
Epithelium
Lamina Propria
100 µm
1 mm
Cyp7b1
Gnai1
Igfbp2
Neat1
Sox4

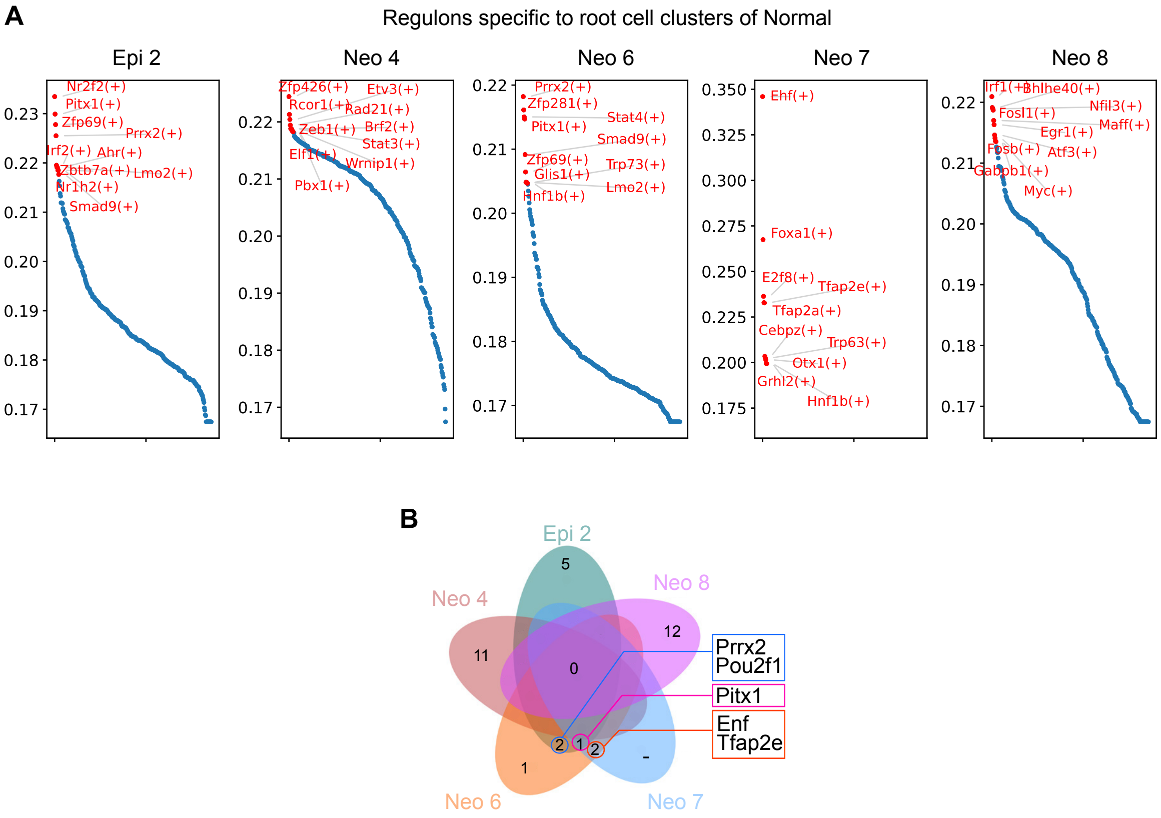
A
Regulons specific to root cell clusters of Normal
Epi 2
Nr2f2(+)
Pitx1(+)
Zfp69(+)
Prrx2(+)
Irf2(+)
Ahr(+)
Zbtb7a(+)
Lmo2(+)
Nr1h2(+)
Smad9(+)
Neo 4
Zfp426(+)
Etv3(+)
Rcor1(+)
Rad21(+)
Zeb1(+)
Brf2(+)
Stat3(+)
Elf1(+)
Wrnip1(+)
Pbx1(+)
Neo 6
Prrx2(+)
Zfp281(+)
Stat4(+)
Pitx1(+)
Smad9(+)
Zfp69(+)
Trp73(+)
Glis1(+)
Lmo2(+)
Hnf1b(+)
Neo 7
Ehf(+)
Foxa1(+)
E2f8(+)
Tfap2e(+)
Tfap2a(+)
Cebpz(+)
Trp63(+)
Otx1(+)
Grhl2(+)
Hnf1b(+)
Neo 8
Irf1(+)
Bhlhe40(+)
Nfil3(+)
Fosl1(+)
Maff(+)
Egr1(+)
Fosb(+)
Atf3(+)
Gabpb1(+)
Myc(+)
B
Epi 2
Neo 8
Neo 4
Neo 6
Neo 7
Prrx2
Pou2f1
Pitx1
Enf
Tfap2e
